# Modulation of Alzheimer’s Disease Aβ40 Fibril Polymorphism by the Small Heat Shock Protein αB-Crystallin

**DOI:** 10.1021/jacs.4c03504

**Published:** 2024-07-08

**Authors:** Natalia Rodina, Simon Hornung, Riddhiman Sarkar, Saba Suladze, Carsten Peters, Philipp W. N. Schmid, Zheng Niu, Martin Haslbeck, Johannes Buchner, Aphrodite Kapurniotu, Bernd Reif

**Affiliations:** ^†^Bayerisches NMR Zentrum (BNMRZ) at the Department of Biosciences, School of Natural Sciences^††^Center for Functional Protein Assemblies (CPA), Department of Biosciences, Technische Universität München, Lichtenbergstr. 4, Garching 85747, Germany; ‡ Helmholtz-Zentrum München (HMGU), Deutsches Forschungszentrum für Gesundheit und Umwelt, Institute of Structural Biology (STB), Ingolstädter Landstr. 1, Neuherberg 85764, Germany; § Division of Peptide Biochemistry, TUM School of Life Sciences, Technical University of Munich, Emil-Erlenmeyer-Forum 5, Freising 85354, Germany; ∥ School of Pharmacy, Henan University, Kaifeng, Henan 475004, China

## Abstract

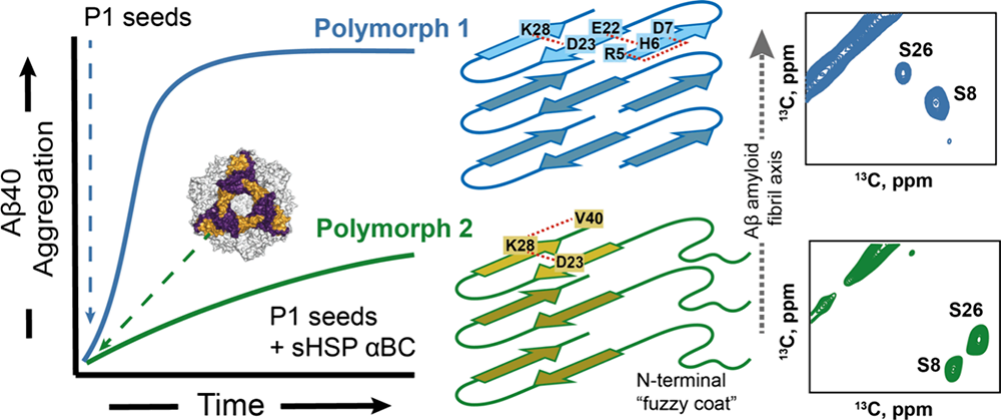

Deposition of amyloid plaques in the brains of Alzheimer’s disease (AD) patients is a hallmark of the disease. AD plaques consist primarily of the beta-amyloid (Aβ) peptide but can contain other factors such as lipids, proteoglycans, and chaperones. So far, it is unclear how the cellular environment modulates fibril polymorphism and how differences in fibril structure affect cell viability. The small heat-shock protein (sHSP) alpha-B-Crystallin (αBC) is abundant in brains of AD patients, and colocalizes with Aβ amyloid plaques. Using solid-state NMR spectroscopy, we show that the Aβ40 fibril seed structure is not replicated in the presence of the sHSP. αBC prevents the generation of a compact fibril structure and leads to the formation of a new polymorph with a dynamic N-terminus. We find that the N-terminal fuzzy coat and the stability of the C-terminal residues in the Aβ40 fibril core affect the chemical and thermodynamic stability of the fibrils and influence their seeding capacity. We believe that our results yield a better understanding of how sHSP, such as αBC, that are part of the cellular environment, can affect fibril structures related to cell degeneration in amyloid diseases.

## Introduction

Aggregation of β-amyloid peptides (Aβ) into amyloid fibrils in the brain tissue is one of the hallmarks of Alzheimer’s disease.^1^ Despite their general overall similarities, Aβ fibrils are known to be highly polymorphic.^2−5^ It is thought that differences in fibril morphology have implications with regard to fibril-induced cellular cytotoxicity, and it has been shown that certain Aβ aggregate morphologies are more toxic in comparison to others.^6,7^ Recently, two structurally defined Aβ polymorphs were shown to promote different pathological changes in susceptible mice.^8^ The occurrence of distinct fibril morphologies is a consequence of flat energy surface of the protein misfolding landscape.^9,10^ We show here that small variations of the solution conditions can influence the conversion into one or the other polymorphic structure.

It is known that the cellular environment influences the kinetics of fibril formation. For example, membranes modulate amyloid fibril growth and can either accelerate or inhibit aggregation.^11−14^ Similarly, glycosaminoglycans such as heparan, keratan, or chondroitin sulfates induce an accelerated conversion into amyloid fibril structures and amyloid aggregates are found to be colocalized in the proteoglycan matrix.^15−17^ Finally, chaperones inhibit protein aggregation and can rescue cells from the cytotoxic side effects of amyloid aggregation.^18−22^ Despite the fact that the cellular context has a direct impact on the kinetics of amyloid formation, it is not understood whether and in which way fibril morphology is changed and how the cellular environment triggers these morphological changes. In this manuscript, we address the question of whether and how the cellular environment can affect amyloid fibril structure. As an example, we focus on the small heat shock protein (sHSP) αB-Crystallin (αBC), which is one of the most intriguing chaperones known to inhibit the aggregation of various amyloidogenic proteins, including Aβ. αBC is upregulated in Alzheimer’s disease, dementia with Lewy bodies and Parkinson’s disease and is found to be colocalized with the amyloid plaques of Alzheimer’s patients.^23,24^ At the same time, it has been shown that αBC modulates Aβ-induced cytotoxicity.^25−28^

Amyloid fibrils are formed via distinct mechanisms and pathways such as primary nucleation, elongation, secondary nucleation, fragmentation etc.^29−32^ Secondary nucleation was found to be the major pathway for the generation of new Aβ amyloid fibrils in which nucleation of Aβ peptide happens on the surface of existing Aβ fibrils.^31,33,34^ The role of secondary nucleation in the structural polymorphism of fibrils is highly debated. It was suggested that secondary nucleation is highly dependent on environmental conditions and can not ensure the preservation of the seed structure in comparison to elongation.^35−37^ On the other hand, preservation of the polymorph structure has been observed in secondary nucleation dominated Aβ aggregation.^38^ In this context, the role of the so-called fibrillar fuzzy coat, i.e. the dynamic residues in the protein sequence that are not part of the fibril core, is not well understood.^39,40^ Interestingly, secondary nucleation and elongation in Aβ aggregates occur at different sites.^41^ Inhibitors can thus differentially interfere with the various aggregation pathways. For example, clusterin is capable of suppressing elongation in Aβ fibrils, while Brichos was found to inhibit secondary nucleation processes and prevent the formation of toxic oligomers.^41,42^ Brichos can bind to Aβ42 fibrils and effectively prevent fibril-catalyzed nucleation even at low concentrations.^43^ Hsp27 and Hsp70 inhibit elongation of α-synuclein fibrils.^44,45^ DnaJB6 binds to Aβ42 fibrils and inhibits secondary nucleation.^46^ αBC interacts with the mature Aβ and α-synuclein fibrils and inhibits their elongation.^47−49^ αBC uses different interfaces to interact with amorphous or amyloid-forming substrates.^50^ Amorphous clients bind to the NTR of the sHSP while Aβ fibrils interacts rather with the edge groove (β4/8) of αBC. Also αBC prevents aggregation at substoichiometric concentrations. It has therefore been hypothesized that αBC binds to fibril ends. It seems thus plausible that the environment influences or even dictates the fibril growth mechanism and redirects amyloids into a distinct polymorphic structure.

In this manuscript, we show how αBC affects Aβ40 fibril polymorphism. Structural changes are probed using MAS solid-state NMR. These experiments are complemented by biophysical assays in which the thermodynamic stability of Aβ40 fibril as well as their seeding competence is probed. In addition, the effects of different fibril polymorphs on the viability of cultured PC12 cells are determined using the MTT reduction assay.

## Results and Discussion

### αBC Inhibits the Replication of the Seed Structure and Leads to the Formation of a New Aβ40 Polymorph

**Scheme 1:**
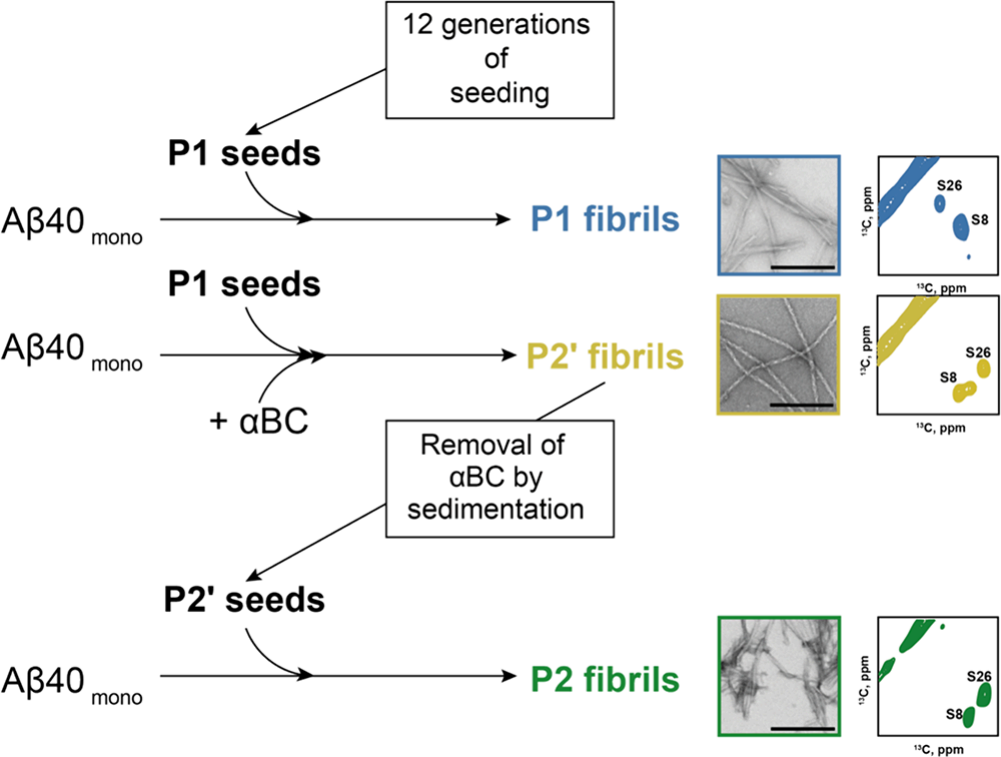
Preparation of Different Aβ40 Polymorphs and Seeds P1 seeds were obtained using a protocol involving 12 generations of seeding, which starts from monomeric Aβ40. The P1 fibril sample was obtained from monomeric ^13^C,^15^N labeled Aβ40 (50 μM) in the presence of 5% P1 seeds. The P2′ fibril sample was obtained from monomeric ^13^C,^15^N labeled Aβ40 (50 μM) in the presence of 5% P1 seeds and 5 μM αBC. P2″ fibrils from Figure S1(not shown in the scheme) were obtained from monomeric ^13^C,^15^N labeled Aβ40 (50 μM) in the presence of 5% P1 seeds and 25 μM αBC. Non-bound αBC was washed away from P2′ fibrils via 5 subsequent rounds of sedimentation (21,000 rcf, 30 min). The pellet was resuspended and sonicated to be used as seeds (P2′ seeds). The P2 fibril sample was obtained from monomeric ^13^C,^15^^13^C,^15^N labeled fibrils. The structure of P1 and P2 fibrils was validated using solid-state NMR. Snapshots of the TEM microphotographs of P1, P2′ and P2 fibril from Figure 1C,D and Figure S2E, respectively are shown. The serine region indicates the different polymorphs from Figure 1E for P1 and P2′, and from Figure 2D for P2 are shown.

**Figure 1:**
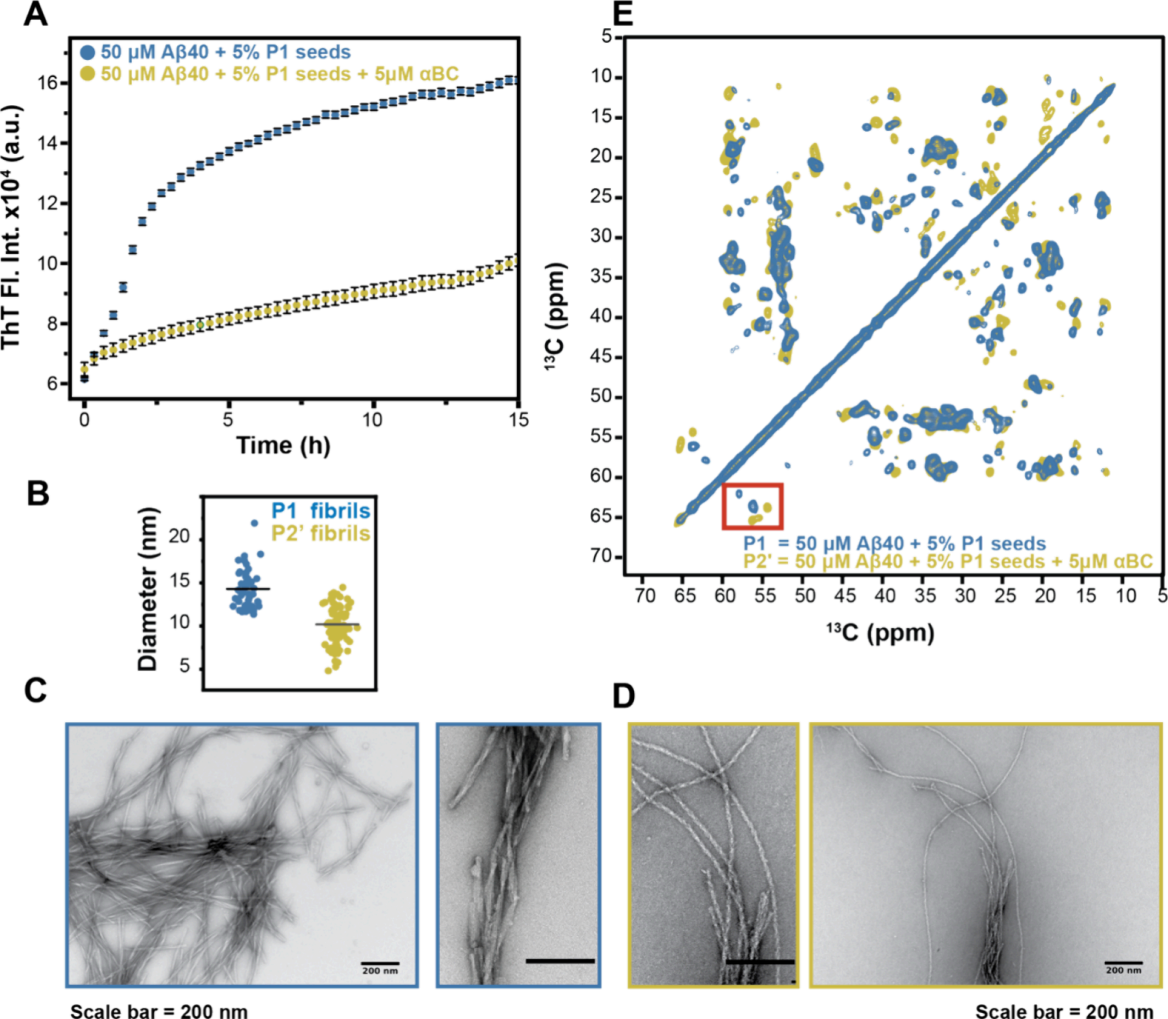
αBC affects seeded Aβ40 fibril formation and structure. (A) ThT kinetic profile of seeded Aβ40 aggregation in absence and presence of αBC. A concentration of 5 μM αBC (yellow) has been employed in the experiment. Means (±SD) of one representative assay (*n* = 3) with *n* = 3 well each are shown. (B) Fibril diameter for polymorph P1 (blue) and P2′ (yellow). The plot shows 50 (for P1) and 100 (for P2′) independent measurements. The horizontal line indicates the mean value. (C) Representative TEM images of Aβ40 fibrils grown using 5% P1 seeds in absence of 5 μM αBC at two magnifications: 60 K on the right and 30 K on the left. (D) Representative TEM images of Aβ40 fibrils grown using 5% P1 seeds in the presence of 5 μM αBC at two magnifications: 60 K on the left and 30 K on the right. (E) Superposition of 2D-^13^C,^13^C MAS correlation spectra of Aβ40 fibrils recorded for samples grown in absence (blue) and presence (yellow) of 5 μM αBC. For all experiments, fibrils were grown using an initial 50 μM monomeric Aβ40 solution. To catalyze fibril formation, 5% P1 seeds have been employed.

To obtain reproducible and well-defined fibril structures, *in vitro* seeded fibril preparations are employed (Scheme 1). Without seeding, heterogeneous solid-state NMR spectra are obtained that show various morphologies in TEM images (Figure S1A,B). In particular, we used a protocol involving 12 generations of seeding carried out at 37 °C.^51^ The Aβ40 fibrils obtained this way are referred to as fibril polymorph 1 (P1) in this work. Although αBC does not seem to completely inhibit fibril-catalyzed seeding, the chaperone induces a dramatic reduction in the aggregation rate and yields a reduction of the ThT plateau intensity (Figure 1A). The amount of insoluble Aβ40 fibrils is reduced and a fraction of Aβ40 peptide remains in solution (Figure S1E). At the same time, the presence of αBC results in a change of fibril morphology as observed in negative stain transmission electron microscopy (TEM) images. This new polymorph is referred to as polymorph 2′ (P2′) (Scheme 1) in the following. In the presence of αBC ([Aβ]:[αBC] = 10:1), P1-seeded fibrils appear more isolated, and less clustered in comparison to the sample that was grown with the chaperone (Figure 1C,D). We have used a molar ration of [Aβ]:[αBC] = 10:1, since this value corresponds best to the physiological situation. The concentration of soluble Aβ in AD brain has been estimated to be in the range of 0.5 to 15 ng per mg of tissue, i.e. (0.1–3.8) nM, while it is on the order of 150 nM in the insoluble fraction.^52,53^ At the same time, the αBC concentration amounts to (140 ± 30) ng per 1 mg of tissue corresponding to a concentration of (7.0 ± 1.5) nM.^54^

In EM images, we observe a range of different diameters in P1 fibrils with an average diameter of 14.3 ± 2.2 nm. The P2′ fibrils formed in the presence of 5 μM αBC appear to be overall thinner with an average diameter of 10.2 ± 1.6 nm (Figure 1B). To characterize the structural differences and properties of the two fibril polymorphs, we prepared fibrils from isotopically labeled Aβ40 for NMR experiments by seeding with P1 in the presence and absence of 5 μM αBC (Scheme 1). To appreciate the homogeneity of our preparations, we recorded carbon–carbon correlation spectra (50 ms mixing time DARR) for both samples. As expected, the fibrils grown with the P1 seeds yield high-quality spectra that contain a single set of NMR resonances (Figure 1E, blue spectrum). Surprisingly, fibrils grown with P1 seeds in the presence of 5 μM αBC (molar ratio Aβ40:αBC = 10:1) and under otherwise identical conditions yield a different cross-peak pattern (Figure 1E, yellow spectrum). Spectral differences for the two preparations are easily identified by inspection of e.g. the serine chemical shift region (highlighted with a red square). The Aβ40 sequence contains two serine residues (S8 and S26). For a homogeneous sample that comprises a single polymorph, exactly two cross peaks should be observed. The differences in cross peak positions suggest that αBC induces a structural change in the fibril morphology.

To verify the influence of αBC on the reproduction of the seed structure, we prepared a second sample (P2″) for which isotopically labeled Aβ40 was seeded with P1 fibrils in the presence of a 5× higher concentration of the chaperone (25 μM αBC) (Figure S1C,D). Under these conditions, the amount of the produced fibrils is heavily reduced (Figure S1E), and thus, the quality of the obtained NMR spectra decreases. Still, the spectral fingerprint is reproduced, and the trend to yield polymorph P2′ is even increased under these conditions (Figure S1C, pink spectrum).

Since a fraction of αBC remains bound to the P2′ and P2″ fibrils after one round of sedimentation (Figure S1E), we wanted to find out whether P2′ morphology requires the presence of the chaperone. For that purpose, several cycles of subsequent sedimentations and resuspensions of the P2′ preparation were performed to separate Aβ40 fibrils and αBC. The obtained fibrils were subsequently sonicated and employed as seeds (Scheme 1). Isotopically labeled Aβ40 seeded with these P2′ seeds resulted in the polymorph P2 (P2). 2D ^13^C,^13^C correlation spectra of P2 fibrils are highly homogeneous and match the spectra of the P2′ preparation (Figure S2A). Interestingly, although the DARR spectra look almost identical and the secondary chemical shifts of P2 and P2′ fibrils are highly correlated (*r* = 0.99) (Figure S2B,C), the overall morphology observed in TEM looks different (Figure S2E). However, the diameter of the individual fibril is unchanged, and is on the order of (9.3 ± 2.4) nm (Figure S2E, inset). The observed differences are due to increased clustering of fibrils which is presumably prevented by αBC in the P2′ preparation. In the following, we were aiming for a more detailed structural characterization of the two Aβ40 fibril polymorphs P1 and P2 (Figure S2D). NMR chemical shift assignments were obtained using 3D NCACX and NCOCX experiments. Chemical shift assignments are deposited in the BMRB under the access code 52337 and 52338, respectively.

### P1 and the αBC-Induced Polymorph P2 Differ in the Structural Order of the Aβ40 N-Terminal Residues

**Figure 2:**
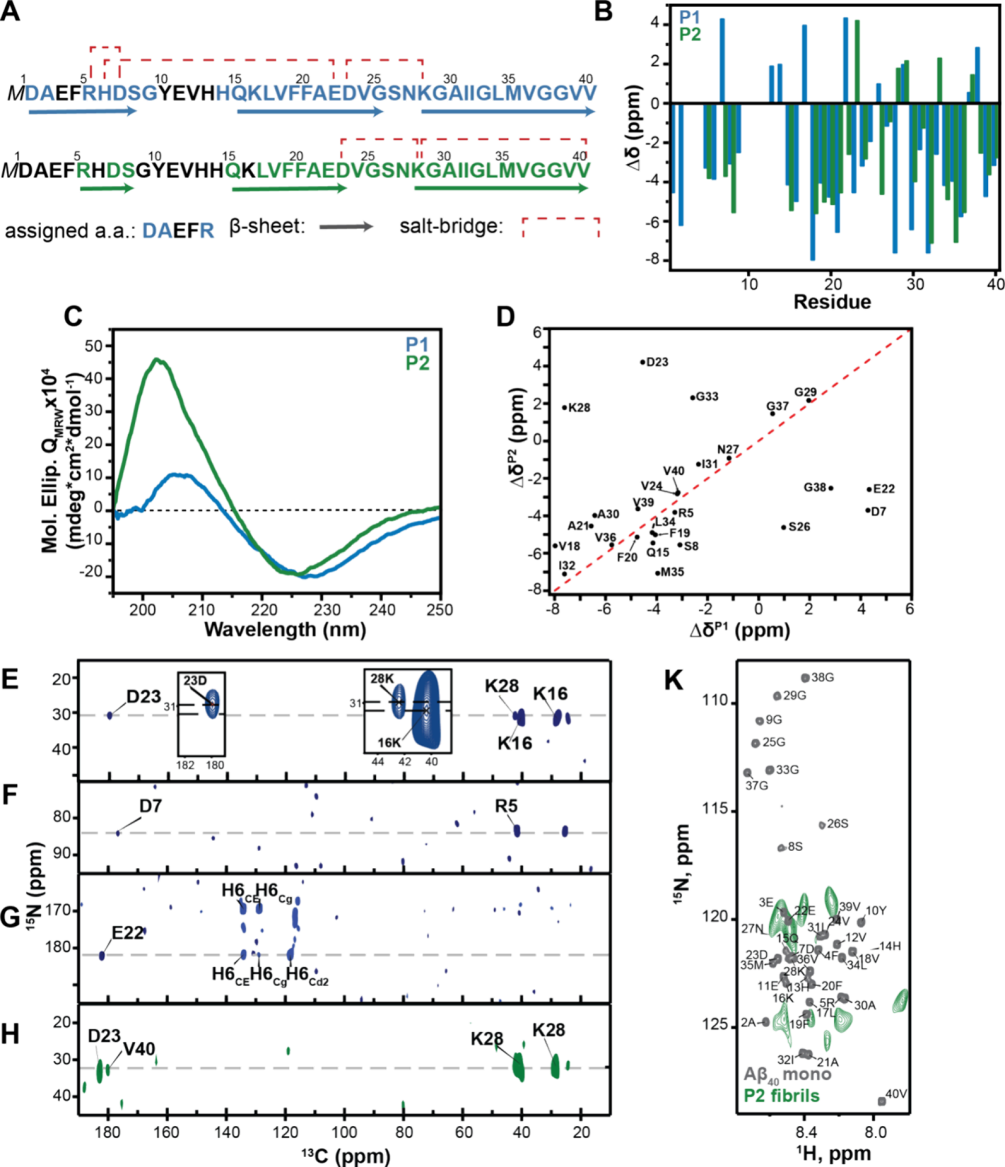
Structural characterization of the two Aβ40 fibrils polymorphs P1 and P2. (A) Amino acid sequence of Aβ40, with assigned residues and beta-sheet secondary structure elements for P1 (blue) and P2 (green). Experimentally observed salt-bridges are represented with red dashed lines. (B) Secondary chemical shifts Δδ for P1 and P2. The overall fibril topology is preserved in the two Aβ40 fibril polymorphs. (C) CD spectra for P1 and P2. The minimum indicative for β-sheet structure is shifted to higher wavelengths (229 nm instead of 225 nm), indicating a more compact structure.^55^ (D) Residue specific secondary chemical shift correlation plot. The *x*- and *y*- axis depict the experimental secondary chemical shifts for P1 and P2, respectively. Expect for residues D7, E22, D23, S26, K28 a high correlation coefficient is observed (*r* = 0.834). The secondary chemical shift is calculated as the differences between the experimentally observed chemical shift and the random coil chemical shift value. (E–G) 2D long-range TEDOR ^13^C,^15^N correlation spectrum recorded for polymorph 1. The selected region shows the salt-bridge involving R5 and D7, K28 and D23, and H6 and E22, respectively. In panel (E), the inset shows that the salt bridge includes a correlation only between K28 and D23, while the ^15^N amino chemical shift of K16 does not match the correlation peak to D23. (H) 2D long-range TEDOR ^13^C,^15^N correlation spectrum recorded for polymorph 2. The selected region shows two salt-bridges involving K28 and D23, K28 and V40. (I) Superposition of scalar coupling based ^1^H,^15^N correlation spectra for Aβ40 monomer in solution (gray) and P2 fibrils in the solid-state (green). The observed P2 chemical shifts are distinct from the resonances obtained in solution suggesting that the chemical environment for flexible residues in the N-terminus of the fibrils is affected by the core. INEPT based correlation spectra recorded for P1 fibrils yield no cross peaks.

To get an estimate of the structural differences between the two polymorphs, we compared the assignable residues in the two preparations. In solid-state NMR experiments, only resonances of rigid residues that are conformationally homogeneous can be observed. While the assignment of P2 only starts from amino acid R5 and has gaps until residue V18, residues D1 and A2, together with amino acids R5 to G9 are observable in polymorph P1 (Figure 2A). The analysis of the secondary chemical shifts reveals that both fibril polymorphs contain 3 β-sheets, however, in the P2 polymorph, the N-terminal β-sheet is truncated compared to P1 (Figure 2A,B). The observed differences between P1 and P2 in NMR chemical shift analysis are accompanied with differences in their secondary structure observed by CD spectroscopy (Figure 2C). In CD we observe a shift of the minimum to larger wavelengths for P1 in comparison to P2 fibrils which could be due to a better-defined structure.^55^ The secondary chemical shifts of P1 and P2 fibrils are rather similar, with the exception of D7, S8, A21, E22, D23, K28, G33, M35 and G38. The chemical shift correlation plot indicates that these residues are off the diagonal (Figure 2D), suggesting that these residues are key elements that determine the fibril topology and polymorphism.

It has been shown that salt bridges affect fibril polymorphism by stabilizing intermolecular interactions in the fibril.^56^ To better characterize salt bridges in P1 and P2 fibrils, we implemented TEDOR solid-state NMR experiments. We observe three distinct salt-bridges for polymorph P1, in particular, H6-E22, D23-K28 and within the N-terminal residues of Aβ40 involving R5-D7 (Figure 2E–G). The R5-D7 and H6-E22 salt-bridges presumably contribute to the increased stability and the loss of flexibility of the N-terminal residues (1–10) in the P1 fibril polymorph. In the P2 polymorph, the characteristic salt-bridge D23-K28 is present, which is found in most Aβ40 fibril structures, while the salt-bridges involving the N-terminus are missing. By contrast, we find a second salt-bridge between K28 and V40 that potentially induces an additional stabilization of the C-terminus (30–40) in P2 fibrils (Figure 2H). We assume that this salt-bridge has a similar structural function as the characteristic K28-A42 salt-bridge found in Aβ42 fibrils or the K28–V40 salt-bridge found in the Aβ40 “Iowa” mutant.^57,58^ For P2, the cross peak representing the D23-K28 salt-bridge is very weak in comparison to P1 (Figure S3A,B), suggesting that the salt bridge interaction in P2 is presumably dynamic. Although P1 and P2 share a similar fibril core structure, the two polymorphs differ in the flexibility of the N- and C-terminal regions. To further characterize differences in dynamics between P1 and P2 fibrils, we recorded INEPT based experiments (Figure 2K and Figure S3C). P1 fibrils do not yield any resonances in case scalar coupling-based transfer elements are employed, suggesting that the structure does not contain dynamic residues (Figure S3C, blue). At the same time, P2 fibrils yield a few weak INEPT cross peaks. For P1 and P2 fibrils, the core parts (17–40) are highly similar. Both fibrils contain a loop region involving residues 10–14. We do not expect any INEPT resonances from these loop residues since dynamics in this region is restricted due to the neighboring β-sheets. The appearance of INEPT resonances in P2 fibrils must thus be due to the dynamic N-terminal region. The ^1^H,^15^N correlation spectra for monomeric Aβ40 in solution and P2 fibrils in the solid state are unlike, suggesting that the chemical environments are distinct. The overall sensitivity of the INEPT based experiments prevents the identification of individual amino acids and a sequential assignment.

The role of the flexible parts of the fibrillar structure was neglected for many years. However, recently it became evident that the so-called “fuzzy coat” of the fibrils plays a crucial role in the process of fibril formation (especially elongation and secondary nucleation), and is an important driving force for intermolecular interactions, e.g. with membranes, mRNA and chaperones.^37,59,60^ The cryo-EM structures of Aβ42 filaments from human brains in familial AD have an extended fuzzy coat (residues 1–11) compared to the filaments found in sporadic AD (residues 1–8) and differ as well in the packing of the protofilament.^61^ A detailed comparison of these polymorphs suggests that G33 and G38 are involved in an interaction with the N-terminus fuzzy coat that shields the hydrophobic C-terminus from the solvent.^62^ Our experimental results indicate that P1 fibrils lack this N-terminal fuzzy coat, which potentially has consequences for the interactions of fibrils with their environment.

**Figure 3:**
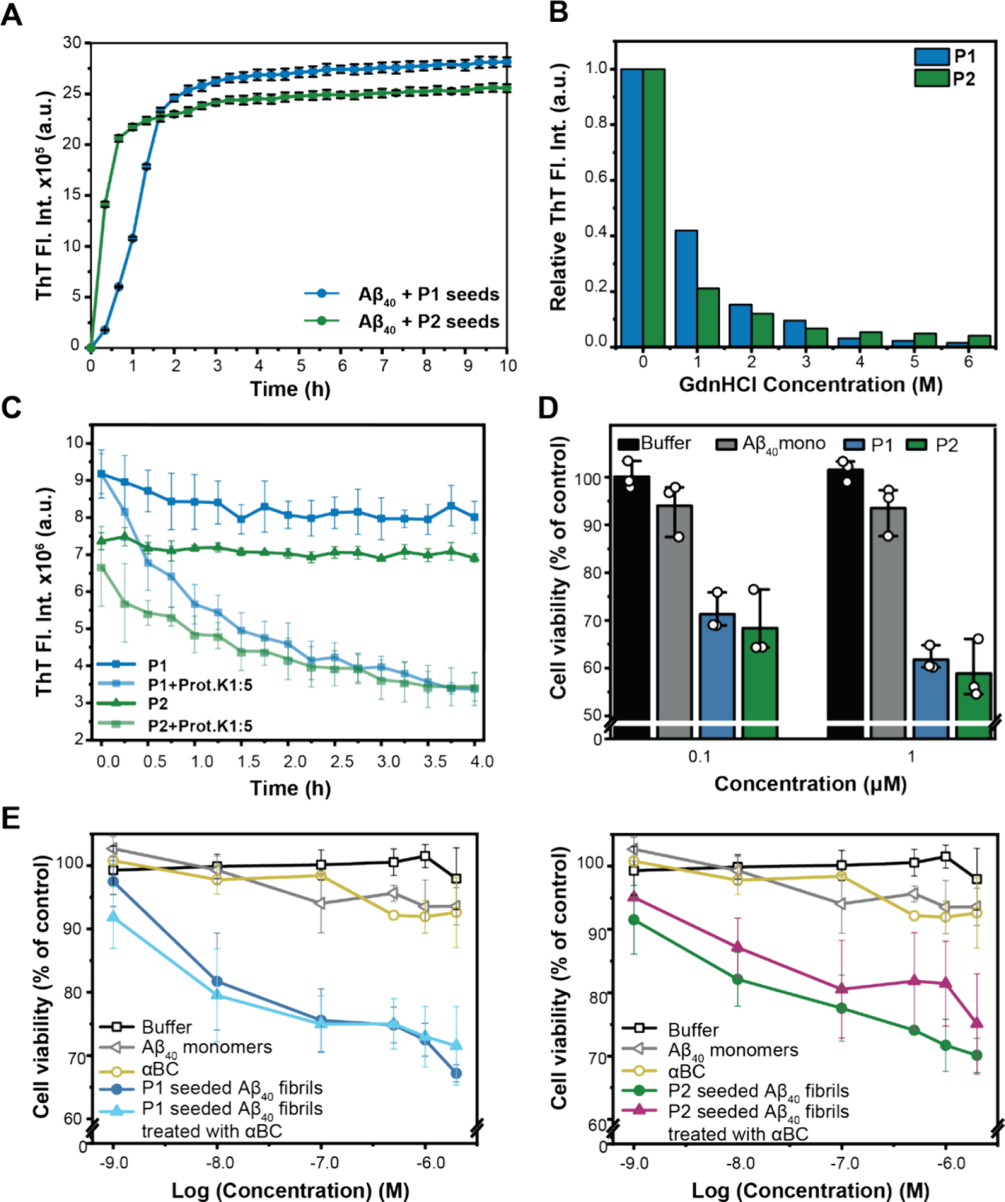
Functional characterization of Aβ40 fibril polymorphs P1 and P2. (A) ThT aggregation profile of seeded Aβ growth using P1 (blue) and P2 (green) seeds, respectively. P1 seeds catalyze fibril formation more efficiently, while P2 seeds yield a sigmoidal seeding kinetics which is characteristic for an activated growth mechanism. The experiment was performed in triplicates. Averaged data is shown. The error bar reflects the standard deviation for the ThT fluorescence values of the triplicates at each time point. (B) GdnHCl disaggregation assay. Representative normalized ThT fluorescence intensity from 2 assays is represented as a function of the GdnHCl concentration. At low GdnHCl concentrations, P1 fibrils are more resistant to chemical denaturation, while at high GdnHCl concentrations P2 fibrils are more stable. (C) Proteinase K digestion assay to probe the stability of Aβ40 fibril polymorph 1 (blue) and polymorph 2 (green). Not normalized ThT fluorescence intensity in absence (dark blue/green) and presence of proteinase K (light blue/green) is represented. A molar ratio [proteinase K]:[Aβ40] = 1:5 has been used in the experiment. The experiment was performed in triplicates. Averaged data is shown. The standard deviation for the fluorescence values of the triplicates is shown as error bars. The normalized data shown in Figure S4A. (D) Cell viability of cultured PC12 cells after treatment with 0.1 μM and 1 μM Aβ40 fibril polymorph 1 (blue) or polymorph 2 (green) determined by MTT reduction assay. The viability of PC12 cells treated with buffer and Aβ40 are shown as controls. Data are shown as means (±SD), 3 assays with *n* = 3 wells each. The individual assays 1–3 are shown in Figure S4C. (E) Cell viability of cultured PC12 cells after treatment with Aβ40 fibrils seeded with P1 (left) or P2 (right). Mature P1 and P2 seeded fibrils were incubated with 5 μM αBC for 1 h prior to the MTT reduction assay. The viability of PC12 cells treated with buffer, αBC and freshly dissolved Aβ40 monomers are shown as controls. Data are shown as means (±SD) of 2 (for fibrils with and without treatment with αBC and for αBC alone) or 3 (for buffer and Aβ40 monomers) assays with *n* = 3 wells each). For clarity, data for P1 and P2 seeded fibrils are shown separately in the left and right panels, respectively.

In our experiments, both P1 and P2 polymorphs can be reproduced via seeding through a secondary nucleation-dominated mechanism (Figure 3A). Similarly, unseeded Aβ40 monomers prepared under identical conditions fibrillize following a secondary nucleation mechanism (Figure S1D). At the same time, as already mentioned before, solid-state NMR shows that the resulting fibrils are heterogeneous (Figure S1A). Surprisingly, only 2 sets of serine peaks corresponding to P1 and P2 could be identified. This implies that both structures can be induced and are consistent with the environmental conditions.

The seeded ThT aggregation kinetics in the presence of αBC implies that Aβ fibrils rather grow by an elongation-dominated mechanism. It is assumed that different sites in Aβ aggregates are responsible for secondary nucleation and elongation.^41^ The question of the role of the environment and its influence on the fibril structure is, however, still debated.^63−65^ While the Buell group suggests that elongation preserves the seed structure, Linse and co-workers have shown that Aβ42 fibril strain characteristics can be efferently propagated in secondary nucleation-dominated systems.^34−36,38^ In our case, the P1 structure is not propagated in the presence of the chaperone, although the solvent and aggregation conditions are identical. The absence of the lag-phase in the seeded aggregation experiments in the presence of the sHSP indicates that the chaperone does not completely inhibit seeding (Figure 1A and Figure S1D). The action of αBC prevents the generation of a compact fibril structure and the conversion of the full amino acid sequence into an amyloid fibril. The appearance of a new polymorph with a dynamic N-terminus in the presence of αBC seems to indicate that the chaperone directly interacts and destabilizes the Aβ40 N-terminal residues in the fibril structure and changes the microenvironment in the nucleation process, resulting in a loss of preservation of the fibril structure.

### Increased Flexibility of the N-Terminus Leads to Changes in Fibril Stability and Seeding Properties

Amyloid fibrils are known to be polymorphic, but a link between the fibril structure and its cellular properties is yet to be identified. Obviously, differences in fibril structure have a direct impact on the respective protein misfolding disease and their progression. Initially, it was believed that bypassing primary nucleation through seeding allows to replicate the seed structure.^66−68^ However, the ability of mature fibrils to act as seeds and templates for the propagation of their structure can differ significantly. E.g., it has been shown recently that *ex vivo* fibril material extracted from patients is often incapable to template and propagate its structure *in vitro.*(5,69,70)

To better understand the fibril polymorph properties, we tested the capacity of the different polymorphs to act as seeds. In particular, we addressed the question of how efficiently the different polymorphs are able to propagate their structures. Our solid-state NMR experiments showed that both polymorphs P1 and P2 can reproduce their structures upon seeding (Figure S2D). In ThT experiments, we find that P2 seeds are able to catalyze Aβ40 fibril formation faster and more efficiently in comparison to P1 seeds (Figure 3A). In both experiments, the same amount of seeds has been employed. We hypothesize that the proper arrangement of the Aβ40 N-terminus in the P1 amyloid fibril is time-limiting in the fibril growth kinetics. Interestingly, seeding with P1 or P2 using the same concentration of Aβ40 monomer results in a higher ThT plateau fluorescence intensity for the P1 seeded fibrils. A high ThT fluorescence quantum yield is related to the inhibition of rotations around the bond connecting the benzothiazole and benzylamine rings of the molecule.^71−73^ It was suggested, that clustering of fibrils results in additional binding sites with a higher fluorescence quantum yield.^74−76^ In these studies, the CD spectra of the insulin fibrils showed a red wavelength shift compared to the lysozyme fibrils which suggested a correlation between clustering of individual fibrils and ThT binding. A higher fluorescence intensity in P1 fibrils could thus be a consequence of an increased rigidity of ThT in the bound state due to fibril clustering or might result from an enlarged number of binding sites (such as β-sheet structures) in the ordered N-terminus of the P1 fibrils.

Next, we examined the chemical stability of the two fibril polymorphs to better understand the fibril properties. Using ThT fluorescence as a read-out, we compared the stability of P1 and P2 fibrils in the presence of different amounts of GdnHCl (Figure 3B). We find that P2 fibrils are less stable when treated with small amounts of GdnHCl (up to 3 M). ThT is considered to specifically interact with β-sheet structures and binds to fibril grooves.^77,78^ In P2 fibrils, the β-sheet core seems easier accessible for GdnHCl due to the solvent accessible N-terminal fuzzy-coat. In the P1 fibrils, the core structure appears to be better protected from chemical degradation as the GdnHCl has to dissolve first the more stable fibril surface. At high GdnHCl concentrations (4–6 M), however, P1 looses its structure more quickly in comparison to P2. We hypothesize that the Aβ40 N-terminus is already dissolved under these conditions, and GdnHCl starts to attack the C-terminal part of the fibril core. In P2 fibrils, the C-terminal region is stabilized by the extra salt-bridge between K28 and V40, which inhibits further degradation. Our findings are in good agreement with a recent computational study of the Vendruscolo group on two types of human brain-derived Aβ42 fibril polymorphs which show that the fuzzy coat increases the overall solubility of the cross-β core of the filaments.^62^

Similar effects are observed in proteinase K stability assays. Although, as discussed earlier, the initial absolute ThT fluorescence differs for the two polymorphs (for the same concentration of fibrils), the ThT fluorescence plateau value after treatment with proteinase K is identical for the two polymorphs (Figure 3C). This suggests that proteinase K is able to digest the different polymorphs to the exact same final amount. At the same time, inspection of the normalized curves (Figure S4A) and effects of different [Aβ40]:[Proteinase K] ratios (Figure S4B) suggest that P1 and P2 are similarly stable against enzymatic degradation in the first 15 min of treatment. However, after the initial 15 min, P1 fibrils rapidly lose ThT fluorescence intensity.

Many factors affect the cytotoxicity of amyloid fibrils. In addition to the topology of the protofilament, the oligomeric arrangement of the protofilaments in the mature fibrils as well as the fibril length matter.^79−82^ We find that the two polymorphs have similar toxic effects on cultivated PC12 cells (Figure 3D and Figure S4C). The fibril structure and especially intramolecular interactions have been shown to be crucial for Aβ40 fibril induced neuronal cytotoxicity.^83,84^ Korn et al. have shown that the cytotoxicity of Aβ40 fibrils depends on the contact between F19 and L34. We performed CHHC and proton assisted recoupling (PAR) solid-state NMR experiments with various mixing times in order to get information about long-range contacts. Although we could identify the F19 spin-systems in the aromatic region of the 50 ms dipolar assisted rotational resonance (DARR) spectrum of P1, we did not observe any cross peaks to L34 in the 250 ms CHHC experiment (Figure S3C). We were not able to assign any carbon atoms of the F19 ring in the 50 ms DARR spectrum recorded for P2 (Figure S3D). We assume that the contact between L34 and a phenylalanine residue in the amyloid hydrophobic core is too dynamic in our preparations. Korn et al. lyophilized their fibril preparation before packing it into the MAS solid-state NMR rotor. Removal of excess solvent might allow to reduce dynamics and stabilize the fibril structure. Fibrils are maintained in an aqueous environment inside the rotor in our preparations by sedimenting the sample directly into the MAS rotor. The preparation procedure might thus explain these differences in the spectra.

To further investigate the role of the N-terminal fuzzy coat on cell viability and interaction with αBC, we incubated mature fibrils with the sHSP. Our results indicate that treatment of mature P1 fibrils with αBC does not influence their effects on PC12 cell viability, while P2 fibrils that were treated with the same amount of chaperone show a somewhat reduced cell-damaging effect (Figure 3E). The effect on cell viability upon αBC treatment of mature P2 fibrils suggests that interaction of the chaperone with the N-terminal fuzzy coat, which might be responsible for cell penetration and cell membrane disruption, might affect fibril toxicity. P1-induced cytotoxicity is less affected by the chaperone due to the lack of the fuzzy coat. Electrostatic interactions, along with specific interactions with various cell receptors such as integrin, were suggested to play an important role in the interaction of Aβ with cell membranes.^85−88^ Various experiments with deletions and mutations in the N-terminus verified that the N-terminus plays not only an important role in Aβ fibrillation but also impacts on cellular toxicity.^89,90^ In Narayanan et al., we have speculated that the Aβ aromatic hydrophobic core region contributes mostly to the interaction with αBC.^91^ In the analysis of the saturation transfer difference NMR spectroscopy (STD) experiments, however, a potential shift of equilibrium between different Aβ aggregation states induced by αBC has not been taken into account.

Oligomer-induced cytotoxic effects have been suggested to be highly relevant for disease pathogenesis as well.^92^ Nevertheless, the effects of fibril formation in the cell and the consequences on cell fate cannot be neglected.^93−96^ Cells have evolved mechanisms that allow either to degrade fibrils or to reduce the consequences of their presence in organs and tissues.^30,97,98^ Chaperones not only play an important role in preventing aggregation of amyloidogenic proteins and modulate fibril polymorphism but, as we know, interact with mature fibrils.^43,47,48^ Recently, disaggregases have been discovered that are capable to unfold and solubilize amyloid fibrils. All disaggregase machineries known so far such as Hsp104, Hsp40 + Hsp70 + Hsp110, HtrA1 etc. are ATP-dependent.^30,99,100^ Even though that αBC is not a disaggregase and not capable of disassembling an amyloid fibril, small heat shock proteins such human Hsp27 and yeast Hsp26 seem to be able to affect the fibrillar structure and cytotoxicity.^101,102^

It was shown that αBC and CHIP interact with α-synuclein and bind to its unstructured C-terminal domain.^103^ As a consequence, fibril uptake by the cell is diminished. A recent study by Stepananko et al. showed that αBC treatment changes fibril morphology and hints toward degradation of lysozyme and β2-microglobulin amyloid fibrils.^104,105^ Although there is no detailed data on possible disaggregation of amyloid fibrils by αBC, a lot of evidence suggests that the chaperone binds to mature fibrils.^47−49^ One possible mechanism of amyloid-induced cytotoxicity is membrane disruption by shedding of membrane-active oligomers.^106,107^ Tipping et al. demonstrated that chemical cross-linking of β2-microglobulin fibrils with Hsp70 increases the fibril stability and, this way, inhibits leakage of toxic oligomers that cause membrane disruption and cellular dysfunction. In this sense, interactions of αBC with the N-terminal fuzzy coat of P2 might stabilize the amyloid fibril structure and shift the equilibrium from the oligomer to a less toxic fibril state.

## Conclusions

We have shown that the small heat shock protein αBC inhibits propagation of the Aβ40 fibril seed structure and induces the formation of a new fibril polymorph. This polymorph is characterized by a flexible N-terminus and is able to transmit its structure even in the absence of the chaperone. The N-terminal fuzzy coat of the αBC induced fibril polymorph (P2) increases the seeding efficiency and at the same time yields a decrease in the chemical stability at low GdnHCl concentrations. P2 is characterized by a stabilized amyloid core structure which is a consequence of an additional salt-bridge at the C-terminus of the peptide. Although the two polymorphs show similar cytotoxic effects on PC12 cells, P2 induced cytotoxicity seems to be reduced in the presence of αBC. We suggest that the N-terminus of Aβ40 is a key region not only for peptide aggregation but for its interaction with sHSPs as well. Our study sheds light on the molecular origin of fibril polymorphism and contributes to the understanding of the fibril fuzzy coat and its interactions with the cellular environment.

## Supporting Information Available

Description of the experimental procedures, materials and methods; NMR spectra of nonseeded fibril preparation, P1, P2′, P2″ and P2 fibrils; comparative analysis of P2 and P2′ chemical shifts; TEM images of P2 fibrils; long-range contacts in P1 and P2 fibrils; ThT aggregation assay; Proteinase K stability assay; MTT assay (PDF)
