# Supplementary material for: Modulation of Alzheimer’s Disease Aβ40 Fibril Polymorphism by the Small Heat Shock Protein αB-Crystallin
Source: J Am Chem Soc. 2024 Jul 8;146(28):19077–87. doi: 10.1021/jacs.4c03504 (PMC11258688; doi:10.1021/jacs.4c03504)
Supplement: Supplementary file 1 — ja4c03504_si_001.pdf [file ja4c03504_si_001.pdf]

Supporting Information for the manuscript

Modulation of Alzheimer's disease Aβ40 fibril polymorphism by the small heat shock protein αB-crystallin

Natalia Rodina[1,2], Simon Hornung[3], Riddhiman Sarkar[1,2], Saba Suladze[1], Carsten Peters[4], Philipp W.N. Schmid[4], Zheng Niu[5], Martin Haslbeck[4], Johannes Buchner[4], Aphrodite Kapurniotu[3], and Bernd Reif[1,2]

[1] Bayerisches NMR Zentrum (BNMRZ) at the Department of Biosciences, School of Natural Sciences, Technische Universität München, Lichtenbergstr. 4, 85747 Garching, Germany

[2] Helmholtz-Zentrum München (HMGU), Deutsches Forschungszentrum für Gesundheit und Umwelt, Institute of Structural Biology (STB), Ingolstädter Landstr. 1, 85764 Neuherberg, Germany

[3] Division of Peptide Biochemistry, TUM School of Life Sciences, Technical University of Munich, Emil-Erlenmeyer-Forum 5, 85354 Freising, Germany

[4] Center for Functional Protein Assemblies (CPA), Department of Biosciences, Lichtenbergstr. 4, 5747 Garching, Germany

[5] School of Pharmacy, Henan University, Kaifeng, Henan 475004, China

Experimental section

1. Sample preparations

1.1. Recombinant Aβ40 expression and purification

Competent BL21 (DE3) cells were transformed with the aid of the pET28a(+) (Novagen) vector carrying the DNA sequence coding for the Aβ40 peptide as insert. The transformed cells were grown up to an optical density of ~0.6-0.7 at 600 nm (OD_{600}) either in lysogeny broth (LB) or isotopically enriched M9 medium ($^{15}NH_4Cl$ (0.5 gL^{-1}),^{13}C glucose (2 gL^{-1}), kanamycin (50 mgL^{-1})). The overexpression was carried out for 4 hours at 37°C after addition of 1 mM isopropyl-b-d-thio-galactopyranoside (IPTG) at 130 rpm. In order to prepare the inclusion bodies (IB) containing the Aβ40 peptide, cell pellets - harvested from 1 L culture each - were resuspended in 50 ml 20 mM Tris·HCl buffer (pH 8.0) containing 20 mg*mL^{-1} DNAse I and 2 tablets of complete protease inhibitor (Roche). The cells were lysed with sonication for 5 minutes (30% amplitude, 1 s pulse on, 1 s pulse off). The IBs were further centrifuged for 30 min (24,000 rcf, 4°C) and the obtained pellet was resuspended via sonication (3 min, 30% amplitude, 1 second pulse on, 1 s pulse off) in 20 mM Tris buffer (pH 8.0) containing 0.4% Triton-100 and 1 tablet of complete protease inhibitor. After the second round of centrifugation, the IBs were washed with 20 mM Tris buffer (pH 8.0) containing 1 tablet of complete protease inhibitor and pelleted via centrifugation. To break the IBs, the pellet was resuspended in 20 mM Tris (pH 8.0) buffer containing 6 M GdnHCl and after 10 minutes incubation on ice, sonicated for 3 minutes (30% amplitude, 1 second pulse on, 1 s pulse off). The dissolved IBs were centrifuged for 30 min (24,000 rcf, 4°C) using a fiberlite F21-8×50y fixed-angle rotor (Thermo-Fisher). The supernatant was subsequently filtered through a 0.22 μm MWCO membrane and loaded onto a reveres-phased chromatography SOURCE30 RPC column that was equilibrated using 80% buffer A (10mM NaN_3) and 20% buffer B (80% acetonitrile, 0.3% TFA). A gradient from 20 to 60% of buffer B was applied using a Dionex UltiMate 3000 HPLC system (Thermo Scientific). Aβ40 peptide in the collected fractions was detected by absorbance at 200 nm at a concentration of 40-45% buffer B. The approximate concentration of the eluted peptide in each fraction was determined using a NanoDrop 2000 spectrophotometer (Thermo Scientific). The purity of the peptide was tested using mass spectrometry and SDS-Tris gel electrophoresis. The fractions of the eluted peptide were transferred either to glass vials or Protein LoBind tubes, lyophilized and stored at -80°C.

1.2. Preparation of Aβ40 peptide stock

All Aβ40 stocks were freshly prepared before each experiment and maintained on ice. Lyophilized recombinant Aβ40 was dissolved in 10 mM NaOH to a final concentration of approximately 200 μM. The solution was sonicated in a water bath twice for 3 min, and cooled down on ice between the sonication cycles. To remove pre-aggregated protein, the solution was transferred to Protein LoBind Eppendorf tubes, and centrifuged for 20 min at 21,000 rcf at 4°C. Afterwards, the supernatant was

filtered through a filter with a 0.2 μm MWCO membrane. This was followed by another round of centrifugation for 30 minutes at 21,000 rcf, at 4°C. The concentration of the stock solution was determined from the absorption spectrum recorded in a high precision cell 10 mm quartz cuvette (Hellma Analytics) using a V-750 Spectrophotometer (Jasco, Japan) with extinction coefficient ε_{280} =1,490 $M^{-1} \cdot cm^{-1}$ that was calculated using the ProtParam online tool. [1]

1.3. Recombinant αB-crystallin (αBC)

Recombinant wild-type αB-crystallin (αBC) was expressed and purified as described elsewhere.[2,3] The protein was aliquoted and stored at -80°C. The protein aliquots were thawed on ice before each experiment.

1.4. Preparation of fibril seeds

Seeds were prepared from mature Aβ40 fibrils with known structural characteristics. Initial polymorph 1 (P1) fibril seeds were obtained using a protocol involving 12 generations of seeding as previously described.[4] All fibril samples were grown at 37 °C. Polymorph 2 (P2) fibril seeds were obtained by washing off αBC from P2' fibrils. The sample preparation of P2' is described in the section *"Preparation of ^{13}C,^{15}N labeled Aβ40 fibrils in the presence of αBC (P2')"*. To wash away αBC, the fibrils were centrifuged at 4°C and 21,000 rcf. The supernatants were carefully removed at each step and the pellet was resuspended in the same amount of 50 mM phosphate buffer (pH 7.4, supplemented with 50 mM NaCl, 0.1% NaN_3). The procedure was repeated 5 times. After the last cycle, the fibrils were dialyzed against a fresh buffer at 4 °C overnight. Seeds were prepared directly prior to the seeding experiments by sonicating a small volume of the fibrils in a glass vial for 5-10 minutes in a water bath.

1.5. Preparation of ^{13}C,^{15}N labeled Aβ40 amyloid fibrils

Recombinant Aβ40 peptide (^{13}C,^{15}N-labeled or non-labeled) was prepared as described in section "*Preparation of Aβ40 peptide stock*". The fibrils were grown in glass vials at 37 °C, under constant shaking at 160 rpm (Innova 40, New Brunswick Scientific). Before the start of each preparation, a small aliquot of the sample was taken and ThT (end concentration 10 μM) was added. The effectiveness of seeding and inhibition by αBC was monitored as described in the section "*Thioflavin T aggregation assay*" for each fibril preparation. For all preparations, the mature fibrils were visualized with TEM (section "*Transmission electron microscopy (TEM)*"). The full conversion of Aβ40 from the monomeric state into the fibrillar state was tested by sedimentation, using a small aliquot of the sample and by measurement of the absorbance of the supernatant.

1.5.1. Preparation of ^{13}C,^{15}N labeled Aβ40 fibril polymorph 1 (P1) and polymorph 2 (P2) for solid-state NMR experiments

For the non-seeded preparation, the freshly prepared Aβ40 stock was diluted with 50 mM phosphate buffer (pH 7.4, supplemented with 50 mM NaCl, 0.1% NaN_3) to a final concentration of 50 µM (0.224 mg/ml). The total final amount of peptide was 10 mg. For the seeded preparation (with P1 or P2 seeds), fibrils were prepared via two rounds of seeding. In the first generation, the peptide stock was diluted with 50 mM phosphate buffer (pH 7.4, supplemented with 50 mM NaCl, 0.1% NaN_3) to a final concentration of 50 µM (0.224 mg/ml). Subsequently, 5% (w/w) of seeds were added. The total final amount of labeled peptide in the first generation was 2 mg. Fibrils were incubated for 3 days. The first generation was used as seeds and mixed with a fresh batch of Aβ40 diluted with 50 mM phosphate buffer (pH 7.4, supplemented with 50 mM NaCl, 0.1% NaN_3) to a final concentration of 50 µM. The total amount of ^{13}C,^{15}N labeled Aβ40 in the second round was 10 mg. The second generation of fibrils was grown for 10 days, as described above.

1.5.2. Preparation of ^{13}C,^{15}N labeled Aβ40 fibrils in the presence of αBC (P2')

To study the influence of αBC on Aβ40 polymorphism, fibrils were grown in the presence of the chaperone. For this purpose, fibrils were grown for one generation using P1 seeds, as described in the section "Preparation of *^{13}C,^{15}N labeled Aβ40 fibril polymorph 1 (P1) and polymorph 2 (P2) for solid-state NMR experiments*". In the second round, αBC was added. Two different Aβ40:αBC ratios were tested: 2:1; 10:1. The second generation in the presence of αBC was grown for 10 days as described above.

2. Characterization of fibril growth and their properties

The recombinant Aβ40 stock solution was freshly prepared before each experiment as described in the section "*Preparation of Aβ40 peptide stock*", and maintained on ice. Seeds were prepared as described in the section "*Preparation of fibril seeds*". The fibrils were grown and handled in 50 mM sodium phosphate buffer (pH 7.4) supplemented with 50 mM NaCl and 0.1 % NaN_3 if not mentioned otherwise. Protein LoBind tubes (Eppendorf) were used for all experiments. Half-area 96-well polystyrene plates with non-binding surface (Corning) were used in well-plate assays.

2.1. ThT aggregation assay

Thioflavin T (ThT) powder was bought from Sigma and used without further purification. ThT was dissolved in water to a concentration of 500 µM, ε_{412}= 31,600 $M^{-1}\cdot cm^{-1}$, and stored at 4 °C protected from light. [5] Experiments were carried out in triplicates (single sample = 150 µl) and the samples (total volume = 500 µl) were mixed on ice. For each sample, the necessary components were mixed in the

following order: sodium phosphate buffer; ThT (end concentration: 10 μM); αBC from a high concentration stock (final concentrations from 5 to 50μM); 5-10% seeds (w/w); Aβ40 peptide from the freshly prepared stock (final concentrations from 5 to 60 μM). The mixtures were gently mixed with the pipette and transferred to half-area 96-well polystyrene plates with non-binding surface (Corning). The readings were either taken on a FLUOstar Omega (BMG LABTECH GmbH, Germany) plate reader or on a SpectraMax Id5 (Molecular Devices, USA) plate reader. Depending on the instrument, excitation and emission wavelengths were either 448 nm and 482 nm (FLUOstar Omega reader), or 445 nm and 485 nm (SpectraMax Id5 reader), respectively. In the instruments, samples were incubated at 37 °C. Readings were conducted in 20 min intervals from the bottom of the plate. 30 s double orbital shaking (600 rpm) was performed before each reading. The plates were sealed with Polyester sealing film (Starlab). After the kinetic experiments, the samples were transferred into Protein LoBind tubes and maintained at either 4 °C or RT. The fibrils were further used for various assays described in the sections below. To analyze the obtained kinetic data, representative replicates were averaged and the standard deviation was calculated. For the normalization of the curves, an average plateau value for each kinetic curve was used. In order to obtain information about the dominating aggregation pathway, the AmyloFit Online Tool was used.[6]

2.2. *Circular dichroism (CD)*

Far-UV CD spectra were recorded in the wavelength range of 190-260 nm for all Aβ40 fibril polymorphs (using a peptide concentration of 10-50 μM) in the same buffer in which the fibrils were grown. For the experiments, a 1 mm Quartz SUPRASIL precision cell (Hellma Analytics) was employed using a J-1500 CD spectrophotometer (JASCO Co., Ltd, Japan). The experiments were carried out at 10 °C. CD reference spectra of the 50 mM sodium phosphate buffer (pH 7.4, supplemented with 50 mM NaCl, 0.1% NaN3) were recorded and subtracted from the sample spectra for baseline correction. The obtained curves were processed using the software Spectra Manager Version 2 (JASCO Co., Ltd, Japan).

2.3. *Transmission electron microscopy (TEM)*

Continuous carbon-coated copper grids (Ted Pella, Inc, USA) were glow charged for 30 s under reduced pressure. 5 μl of a 50 μM sample was applied on the grids for 90 s. The grids were subsequently washed with 20 μl ddH_2O to remove phosphate salts. 5 μl of a 2 % uranyl acetate solution was applied for 45 s. Micrographs were obtained using a JEOL 1400 plus microscope (JEOL, Japan) at various magnifications. Micrographs were processed, analyzed, and scaled using ImageJ (National Institute of Health, USA). For the quantification of the fibril diameter, only single filaments were used. In case fibrils were twisted, the diameter was measured in the widest part of the fibril. For each polymorph, at least 50 independent measurements were carried out using several independent sample preparations.

2.4. Guanidinhydrochloride (GdnHCl) stability assay

The mature P1 and P2 fibrils were diluted to 10 μM and sedimented via centrifugation for 30 minutes at 4 °C at 21,000 rcf. The supernatants were carefully removed and the pellets were resuspended in an equal volume of 50 mM sodium phosphate buffer containing various amount of guanidine hydrochloride (from 0 to 6 M) and 10 μM ThT. Control samples of 10 μM ThT in sodium-phosphate buffer with the corresponding amounts of GdnHCl were prepared. All samples were transferred to 96-well plates and ThT fluorescence emission at 485 nm was recorded at 25 °C in either the FLUOstar Omega reader (BMG) or the SpectraMax Id5 reader (Molecular devices). The excitation wavelength were set to 445 nm and 485 nm, respectively. We find that the ThT fluorescence slightly increases in the presence of higher molarities of GdnHCl. The fluorescence of the ThT controls were, therefore, subtracted from the values recorded for the corresponding fibril samples. The obtained ThT fluorescence intensities were normalized to the ThT values in the presence of fibrils that were not treated with GdnHCl for each polymorph separately.

2.5. Proteinase K stability assay

Mature P1 and P2 fibrils were diluted to 40 μM with sodium-phosphate buffer containing ThT (final concentration 10 μM). The fibrils were treated with proteinase K (Roche). The following ratios of fibrils to proteinase K were tested: 1:2.7; 1:5; 1:10. A control sample of fibrils without proteinase K treatment as well as proteinase K in absence of the fibrils were prepared. The samples were immediately transferred to 96-well plates and ThT Fluorescence emission at 485 nm with excitation at 450 nm was recorded at 37 °C in either a FLUOstar Omega reader (BMG) or a SpectraMax Id5 reader (Molecular devices). The measurements were performed in 15 minutes intervals and the samples were gently shaken at 150 rpm between the reads. The experiment was performed in triplicates. The measurements were stopped when the ThT fluorescence reached a stable plateau and no changes were observed for at least1 hour. No significant changes in the fluorescence of ThT in the presence of proteinase K were observed indicating that there is no influence of the protease on the properties of the fluorescent dye.

2.6. Assessment of cell damage via the MTT reduction assay

For the comparison of the cytotoxicity of P1 and P2 fibrils with validated structure, the solid-state NMR material unpacked from the corresponding MAS rotors was used. The material was dissolved in 50 mM phosphate buffer (pH 7.4, supplemented with 50 mM NaCl) and a final concentration of 20 μM kept at 4 °C and used directly for the MTT reduction assays. Aβ40 monomers were freshly prepared as described in section "*Preparation of Aβ40 peptide stock*" to a final stock concentration of 20 μM, and directly for the MTT reduction assays.

To study the effects of αBC on the cytotoxicity of mature fibrils, fibrils with various seeding (P1 or P2 seeds) were prepared as for the ThT assay described in the section "*Thioflavin T (ThT) aggregation assay*". 50 mM phosphate buffer (pH 7.4, supplemented with 50 mM NaCl) without addition of NaN_3 was used since NaN_3 was shown to affect cell viability.[7] The amount of used seeds was 5% (w/w). Solutions applied for the MTT reduction assay were prepared in parallel to incubations monitored by the ThT binding assay, but without addition of Thioflavin T and transferred to LoBind tubes at the end of the ThT binding assay kinetics. Mature amyloid fibrils were diluted to a final concentration of 20 μM with 50 mM phosphate buffer (pH 7.4, supplemented with 50 mM NaCl). To study the effects of αBC on the cytotoxicity of mature fibrils, P1 and P2 seeded fibrils prepared as described above were mixed with 2 μM αBC (10:1 ratio) and incubated at RT for 1 hour. 2 μM αBC in 50 mM phosphate buffer (pH 7.4, supplemented with 50 mM NaCl) as well as 50 mM phosphate buffer (pH 7.4, supplemented with 50 mM NaCl) were used as controls.

Studies on the effects of various fibril polymorphs (P1, P2) were performed using cultured PC12 cells obtained from DSMZ (German Collection of Microorganisms and Cell Cultures) (DSMZ no. ACC 159) and the MTT reductions assay as previously described (e.g. ref 8).[8] Briefly, samples prepared as described above were added to PC12 cells at the indicated final dilutions in cell medium. Following incubation with the cells for ~20 h (37 °C, humidified atmosphere with 5 % CO2) MTT reduction was determined using a Multilabel reader VictorX3 (Perkin Elmer Life Sciences) as previously described.[8,9]

2.7 SDS-PAGE

To visualize the sHSP binding to fibrils grown with 5 % P1 seeds in presence of either 5 μM or 25 μM αBC, the samples were sedimented at RT for 30 minutes at 21,000 rcf in LoBind tubes. The supernatants were carefully removed. The pellets were resuspended in the same amount of 50 mM phosphate buffer (pH 7.4, supplemented with 50 mM NaCl, 0.1% NaN_3). SDS-PAGE analysis was performed on 4-12 % Bis-Tris NuPage gels in NuPage MES SDS running buffer and NuPage LDS sample buffer (Invitrogen).

3. Solid-state NMR spectroscopy

3.1. Sample preparation

The fibril preparation procedure is described in section 1.5.1. Grown fibrils were collected by centrifugation at 21,000 rcf (4°C). The supernatants were removed and stored for future use. 1.9 mm ZrO_2 (Bruker Corporation, USA) magic angle spinning (MAS) rotors were packed by sedimenting ~8 mg of material, using the spiNpack 1.9 mm rotor packing tool (Giotto Biotech) and an ultracentrifuge (Optima L100 XP, Beckman Coulter, USA) at 28,000 x rcf at 12 °C using a SW32Ti swinging bucket rotor. The packed rotors were kept at 4°C.

3.2. Experiments

Two-and three-dimensional solid-state NMR spectra of uniformly ^{13}C,^{15}N-labeled Aβ40 fibril samples were recorded on a Bruker Avance III 750 MHz spectrometer, equipped with a triple-resonance (^{1}H,^{13}C,^{15}N) 1.9 mm MAS probe. The MAS rotation frequency was adjusted to 16.65 kHz. The sample temperature was maintained at 10°C using a cooling gas flow of 550 L/h. High-power proton decoupling ($\omega_{RF}/2\pi$= 100 kHz) was applied during acquisition using SPINAL-64. For the ^{1}H->^{13}C magnetization transfer, cross-polarization was employed. ^{13}C,^{13}C transfers were achieved via PDSD or DARR using a mixing time of 30 or 50 ms.[10] For sequential resonance assignment, conventional 3D NCA/NCO, NCACX and NCOCX experiments were employed.[11,12] Selective coherence transfer between ^{15}N and ^{13}C (aliphatic or CO) was achieved using pulse shapes optimized by Optimal Control. [13,14] The long-range interactions were obtained from long mixing time DARR experiments (200, 400, and 600 ms), as well as PAR experiments (5, 15, and 20 ms).[15] To characterize the salt bridges, 2D ^{13}C,^{15}N TEDOR experiments have been recorded.[16-18] In these experiments, the MAS rotation frequency has been adjusted to 16.5 kHz MAS, using short (1.9 ms) and long (either 13 or 15.0 ms) TEDOR mixing times. For long-range interactions between F19 and L34 CHHC experiments with a mixing time of 250 ms were recorded. [19] Chemical shifts were references to external adamantane.

3.3. Processing and data analysis

All spectra were processed using TopSpin3.5 (Bruker Corporation, USA) and CCPNmr 2.3. Assignments were performed using CCPN 3.1.1 (Collaborative Computational Project for NMR).[20] The assignments for P1 and P2 were uploaded to the BMRB (BMRB-ID: 52337 and 52338), and will be published independently. CCPNmr 2.3 was used for analysis. For visualization of 1D spectra, Mnova 11.0 (Mestrelab) was used.

The secondary chemical shifts were calculated as follows:

$$\Delta\delta = [C_\alpha(observed) - C_\alpha(random\ coil)] - [C_\beta(observed) - C_\beta(random\ coil)]$$

Random coil chemical shifts were predicted using the tab2bmrb tool provided by BMRBJ. The correlation coefficient between random coil corrected chemical shifts between published and our experimental data was calculated as follows

$$correl(x,y) = \frac{\sum(x-\bar{x})(y-\bar{y})}{\sqrt{\sum(x-\bar{x})^2 \sum(x-\bar{x})^2}},$$

where $\bar{x}$, $\bar{y}$ represent average x and average y values.

4. Solution-state NMR experiments

Aβ40 monomer sample was prepared by diluting a freshly dissolved ^{13}C,^{15}N labeled Aβ40 peptide (10 mM NaOH) into 50 mM phosphate buffer (50 mM NaP, 50 mM NaCl, 10 % D_2O, pH 7.4) to a final concentration of 50 μM. The sample was immediately transferred to a Shigemi NMR tube. All spectra were recorded at 10 °C on a 600 MHz Bruker NMR spectrometer, equipped with a z-gradient cryogenic

triple resonance NMR inverse (TCI) probe and a Bruker AV-III console. 1D-^{1}H and 2D-^{1}H,^{15}N HSQC experiments were conducted using a ^{1}H,^{15}N HSQC pulse sequence kindly provided by Dr. Sam Asami (Technische Universität München, Germany). All NMR spectra were processed using TopSpin (Bruker Corporation, USA) and analyzed using CCPN 2.4.2 (Collaborative Computational Project for NMR).

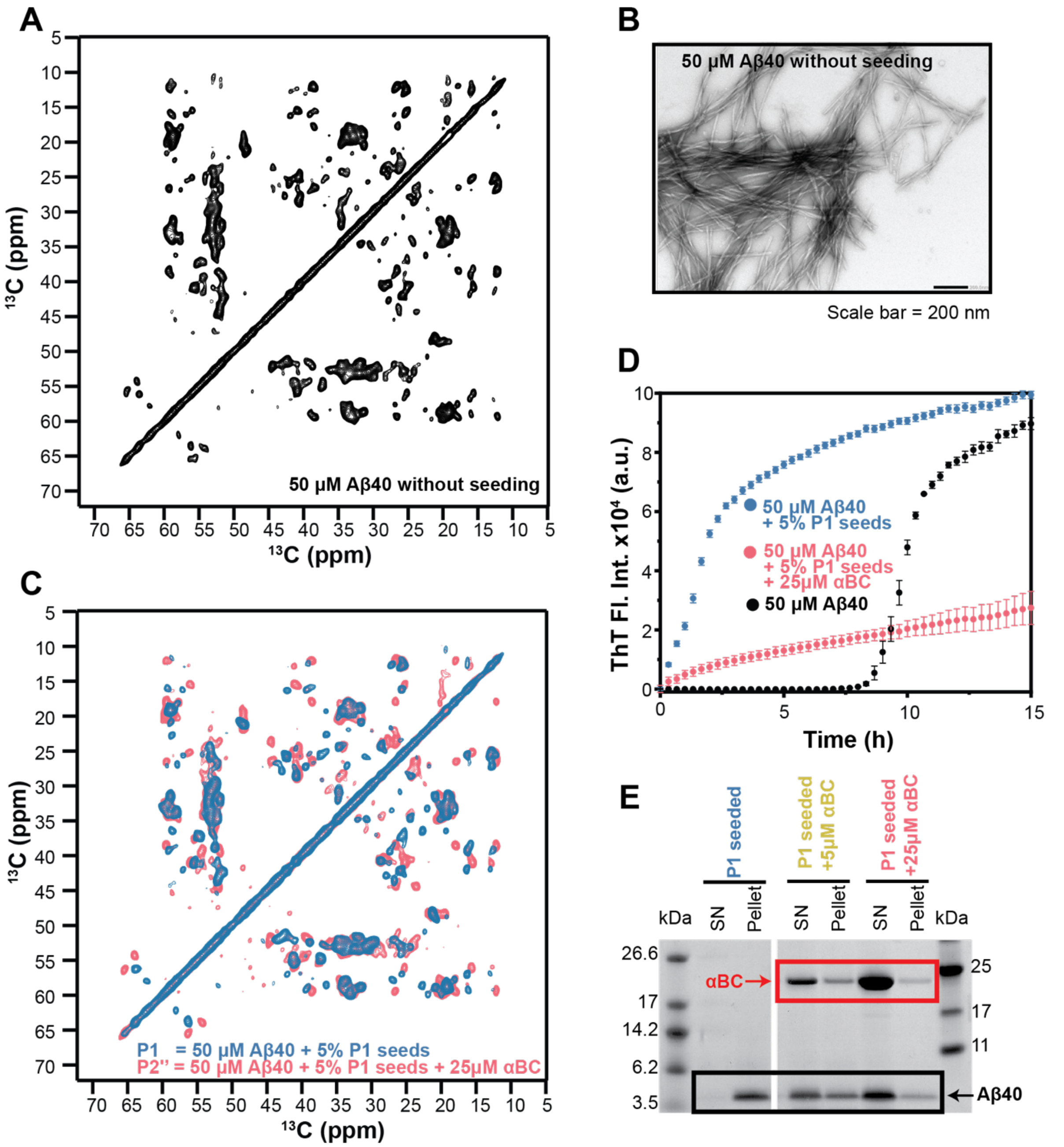

Figure S1. *(A) 2D-^{13}C,^{13}C MAS correlation spectra of Aβ40 fibrils recorded for samples grown in absence of seeds and 5 µM αBC. Fibrils were prepared using an initial 50 µM monomeric Aβ40 solution. (B) Representative TEM image of Aβ40 fibrils grown without seeds. The scale bar corresponds to a length of 200 nm. (C) Superposition of 2D-^{13}C,^{13}C MAS correlation spectra of Aβ40 fibrils recorded for samples grown in absence (blue, P1) and presence of 25µM αBC (pink, P2''). For all experiments, fibrils were prepared using an initial 50µM monomeric Aβ40 solution. To catalyze fibril formation, 5 % P1 seeds have been employed. (D) ThT kinetic profile of non-seeded (black) and seeded Aβ40 aggregation in absence (blue) and presence (pink) of 25 µM αBC. Means (+/- SD) from a representative assay with n=3 is shown. (E) Representative SDS-PAGE analysis of the supernatants and pellets of the fibrillar sample after the ThT aggregation kinetics.*

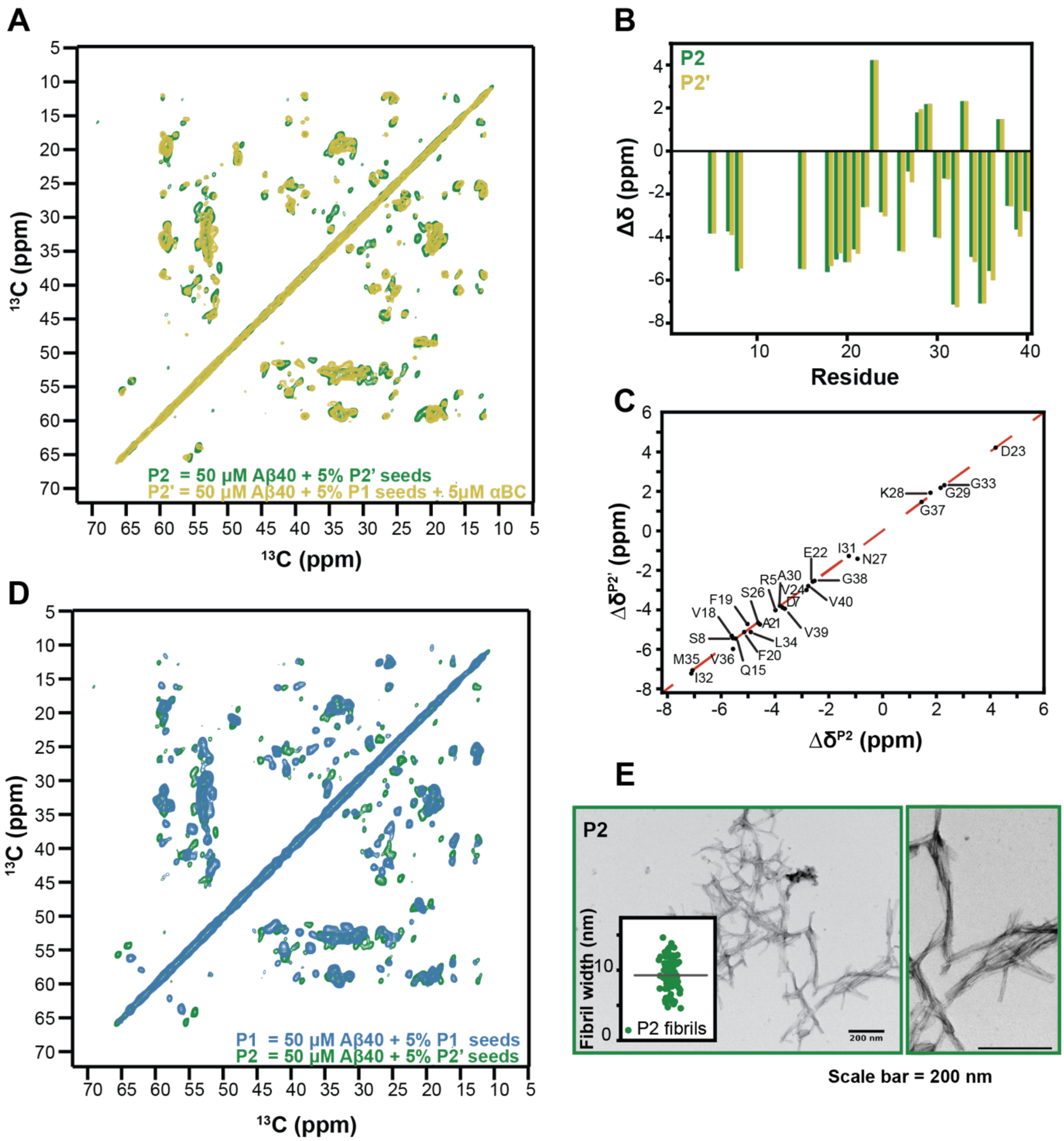

Figure S2. *The P2' structure can be reproduced by seeding in the absence of αBC. (A) Superposition of 2D-^{13}C,^{13}C MAS correlation spectra recorded for P2' (yellow) and P2 (green). P2' was obtained with 5% P1 seeds in presence of 5μM αBC. P2 was obtained using P2' as seeds after washing away αBC. For all experiments, fibrils were grown using an initial 50μM monomeric Aβ40 solution. To catalyze fibril formation, 5 % of seeds have been employed. (B) Secondary chemical shifts Δδ for P2 and P2'. The fibril topology is preserved in the two Aβ40 fibril polymorphs. (C) Residue specific secondary chemical shift correlation plot. The x- and y- axis depict the experimental secondary chemical shifts for P2 and P2', respectively. The secondary chemical shift are highly correlated (r = 0.99), suggesting that the fibril structures of P2 and P2' are identical. The secondary chemical shifts are calculated as the difference between the experimentally observed chemical shifts and the random coil chemical shift values. (D) Superposition of 2D-^{13}C,^{13}C MAS correlation spectra recorded for P1 fibrils (blue) and P2 fibrils (green). For all experiments, fibrils were grown using an initial 50μM monomeric Aβ40 solution. To catalyze fibril formation, 5 % seeds have been employed in both cases. (E) TEM image of Aβ40 fibrils grown using 5% P2' seeds in absence αBC at two magnifications: 60K on the right and 30K on the left. The scale bar corresponds to a length of 200 nm. The insert on the left image shows individual measurements of the P2 fibril diameter. The horizontal line indicates the mean value. In the statistical analysis of the fibril diameter only isolated filaments were used and fibril bundles avoided.*

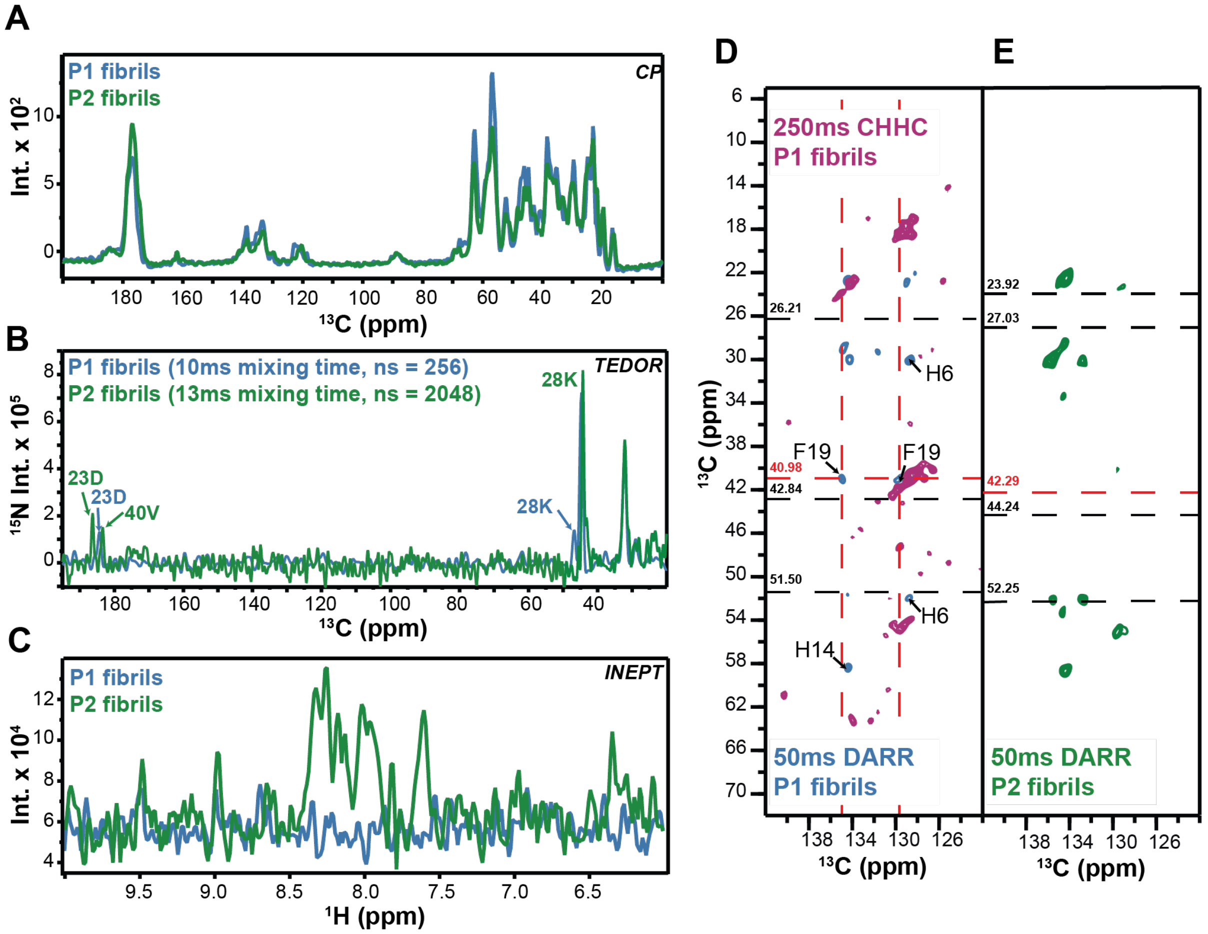

Figure S3. *Long-range contacts in P1 and P2 fibrils. (A) Superposition of 1D-^{13}C CP solid-state NMR spectra of the P1 (blue) and the P2 fibril sample (green). The intensities of the two spectra are rather similar, suggesting that both rotors contain approximately the same amount of sample. (B) Superposition of ^{13}C traces from the 2D ^{15}N,^{13}C TEDOR experiment for P1 (blue, TEDOR mixing time= 10 ms, 256 scans per increment) and P2 (green, TEDOR mixing time= 13 ms, 2048 scans per increment). The traces were extracted at ^{15}N chemical shifts of 30.91 ppm and 31.8 ppm for P1 and P2, respectively. (C) ^{13}C-detected 1D spectra recorded for P1 and P2 fibril samples. In the experiment, an INEPT pulse sequence element was employed for magnetization transfer. (D) 2D ^{13}C,^{13}C correlation spectra focussing on the aromatic spectral region recorded for P1 fibrils. The plot shows a superposition of the 50 ms DARR spectrum (blue) and the 250 ms CHHC spectrum (purple). Peak assignments for H6, H14 and F19 are included. Red lines refer to the resonances of F19. Black lines represent the L34 chemical shifts Cα (51.56 ppm), Cβ (42.84 ppm) and Cγ (26.21 ppm). In the 250 ms CHHC spectrum, no cross-peaks between F19 and L34 are detected. (E) 2D ^{13}C,^{13}C correlation spectrum focussing on the aromatic spectral region recorded for P2 fibrils (green). In the 50ms DARR experiment, no peaks are observable for the F19 spin system. The red line indicates the F19 Cβ resonance frequency. Black lines represent the L34 chemical shifts Cα(51.56 ppm), Cβ (42.84 ppm), Cγ (26.21 ppm) and Cδ (23.92 ppm).*

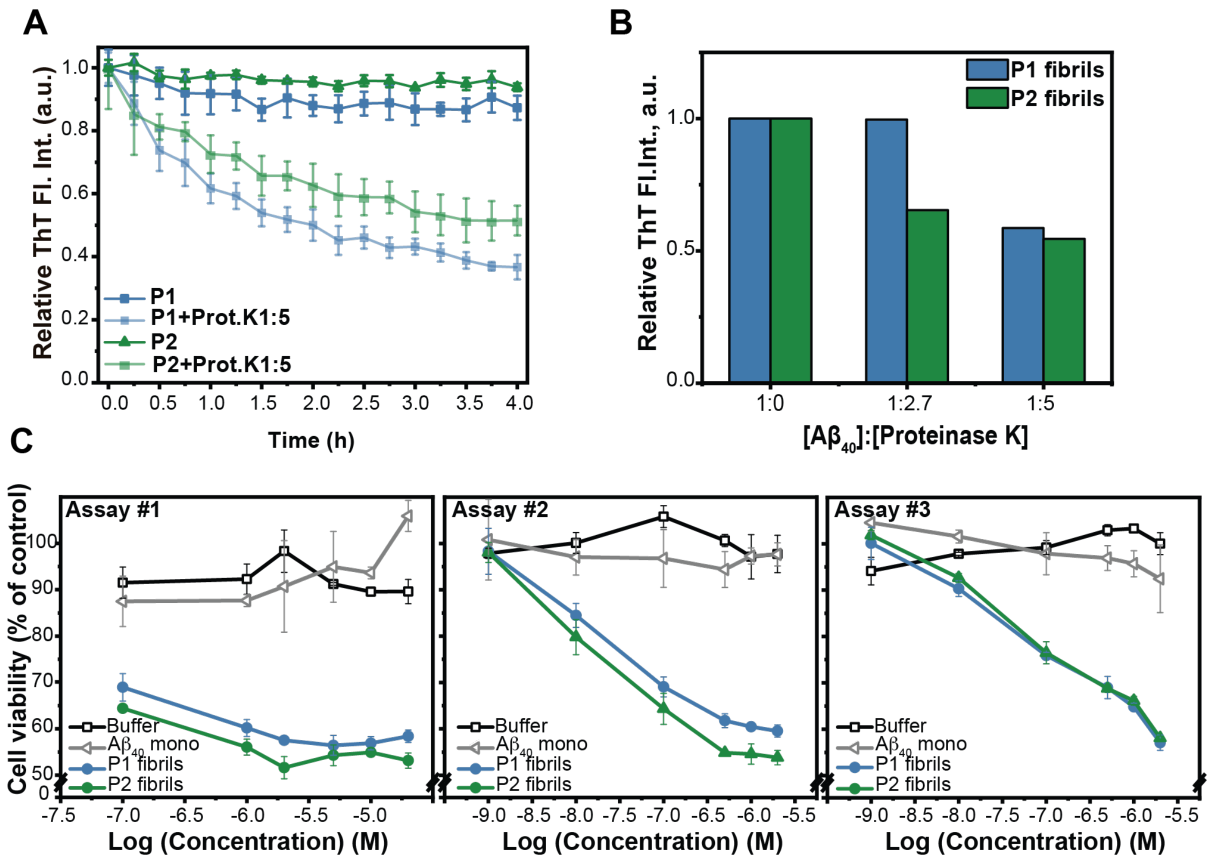

Figure S4. *Cellular toxicity and protease stability assay. (A) Proteinase K digestion kinetic assay to probe the stability of Aβ40 fibril polymorph 1 (blue) and polymorph 2 (green). The panel shows the normalized ThT fluorescence intensity in absence (dark blue/green) and presence of proteinase K (light blue/green). A molar ratio [proteinase K]:[Aβ40]=1:5 has been used in the experiment. The experiment was performed in triplicates. Averaged data is shown. The standard deviation for the fluorescence values of the triplicates is shown as error bars. (B) Proteinase K digestion assay to probe the stability of P1 (blue) and P2 (green) Aβ40 fibrils. The plot represents the normalized ThT fluorescence intensity after 15 minutes of proteinase K treatment. The molar ratios [Aβ40]:[proteinase K]=1:5 and 1:2.7 have been employed for the assay. (C) Concentration-dependence of the effects of P1 and P2 fibrils on PC12 viability. Results from each of the 3 independent MTT reduction assays underlying data shown in Fig. 3D are presented. Fibrils were grown using ^{15}N,^{13}C isotopically labeled Aβ40 with either 5% P1 (blue) or P2' (green) seeds. Prior to the experiment, their structure was validated by solid-state NMR. Data are shown as means (±SD) for each assay, n = 3 wells. In assay #1, a plateau was observed at concentrations above 1μM. Therefore, only lower concentrations were employed in assays #2 and #3.*

References

(1) Gasteiger, E.; Hoogland, C.; Gattiker, A.; Duvaud, S. e.; Wilkins, M. R.; Appel, R. D.; Bairoch, A. In The Proteomics Protocols Handbook; Walker, J. M., Ed.; Humana Press: Totowa, NJ, 2005, p 571-607.

(2) Mainz, A.; Jehle, S.; van Rossum, B. J.; Oschkinat, H.; Reif, B. Large Protein Complexes with Extreme Rotational Correlation Times Investigated in Solution by Magic-Angle-Spinning NMR Spectroscopy. *Journal of the American Chemical Society* **2009**, *131* (44), 15968-15969.

(3) Peschek, J.; Braun, N.; Franzmann, T. M.; Georgalis, Y.; Haslbeck, M.; Weinkauf, S.; Buchner, J. The eye lens chaperone alpha-crystallin forms defined globular assemblies. *Proc Natl Acad Sci U S A* **2009**, *106* (32), 13272-13277.

(4) Lopez del Amo, J. M.; Schmidt, M.; Fink, U.; Dasari, M.; Fändrich, M.; Reif, B. An asymmetric dimer as the basic subunit in Alzheimer's disease amyloid β fibrils. *Angew Chem Int Ed Engl* **2012**, *51* (25), 6136-6139.

(5) Groenning, M.; Olsen, L.; van de Weert, M.; Flink, J. M.; Frokjaer, S.; Jorgensen, F. S. Study on the binding of Thioflavin T to beta-sheet-rich and non-beta-sheet cavities. *J. Struct. Biol.* **2007**, *158* (3), 358-369.

(6) Meisl, G.; Kirkegaard, J. B.; Arosio, P.; Michaels, T. C. T.; Vendruscolo, M.; Dobson, C. M.; Linse, S.; Knowles, T. P. J.; Molecular mechanisms of protein aggregation from global fitting of kinetic models. *Nat Protoc* **2016**, *11*, 252-272.

(7) Slameňová, D.; Gabelová, A.; The effects of sodium azide on mammalian cells cultivated in vitro. **1980**, *71*, 253-261.

(8) Yan, L. M.; Velkova, A.; Tatarek-Nossol, M.; Andreetto, E.; Kapurniotu, A. IAPP mimic blocks Abeta cytotoxic self-assembly: cross-suppression of amyloid toxicity of Abeta and IAPP suggests a molecular link between Alzheimer's disease and type II diabetes. *Angew Chem Int Ed Engl* **2007**, *46* (8), 1246-1252.

(9) Spanopoulou, A.; Heidrich, L.; Chen, H. R.; Frost, C.; Hrle, D.; Malideli, E.; Hille, K.; Grammatikopoulos, A.; Bernhagen, J.; Zacharias, M.; Rammes, G.; Kapurniotu, A. Designed Macrocyclic Peptides as Nanomolar Amyloid Inhibitors Based on Minimal Recognition Elements. *Angew. Chem.* **2018**, *57* (44), 14503-14508.

(10) Takegoshi, K.; Nakamura, S.; Terao, T. C13–1H dipolar-driven C13–13C recoupling without C13 rf irradiation in nuclear magnetic resonance of rotating solids. *The Journal of Chemical Physics* **2003**, *118* (5), 2325-2341.

(11) McDermott, A.; Polenova, T.; Bockmann, A.; Zilm, K. W.; Paulson, E. K.; Martin, R. W.; Montelione, G. T. Partial NMR assignments for uniformly (13C, 15N)-enriched BPTI in the solid state. *J Biomol NMR* **2000**, *16* (3), 209-219.

(12) Pauli, J.; Baldus, M.; van Rossum, B.; de Groot, H.; Oschkinat, H. Backbone and side-chain 13C and 15N signal assignments of the alpha-spectrin SH3 domain by magic angle spinning solid-state NMR at 17.6 Tesla. *Chembiochem* **2001**, *2* (4), 272-281.

(13) Baldus, M.; Petkova, A. T.; Herzfeld, J.; Griffin, R. G. Cross polarization in the tilted frame: assignment and spectral simplification in heteronuclear spin systems. *Molecular Physics* **1998**, *95* (6), 1197-1207.

(14) Tošner, Z.; Sarkar, R.; Becker-Baldus, J.; Glaubitz, C.; Wegner, S.; Engelke, F.; Glaser, S. J.; Reif, B. Overcoming Volume Selectivity of Dipolar Recoupling in Biological Solid-State NMR Spectroscopy. *Angewandte Chemie International Edition* **2018**, *57* (44), 14514-14518.

(15) De Paëpe, G.; Lewandowski, J. R.; Loquet, A.; Böckmann, A.; Griffin, R. G. Proton assisted recoupling and protein structure determination. *The Journal of Chemical Physics* **2008**, *129* (24), 245101.

(16) Hing, A. W.; Vega, S.; Schaefer, J. Transferred-echo double-resonance NMR. *Journal of Magnetic Resonance* **1992**, *96*, 205-209.

(17) Jaroniec, C. P.; Filip, C.; Griffin, R. G. 3D TEDOR NMR Experiments for the Simultaneous Measurement of Multiple Carbon−Nitrogen Distances in Uniformly 13C,15N-Labeled Solids. *Journal of the American Chemical Society* **2002**, *124* (36), 10728-10742.

(18) Lopez del Amo, J. M.; Fink, U.; Dasari, M.; Grelle, G.; Wanker, E. E.; Bieschke, J.; Reif, B. Structural properties of EGCG-induced, nontoxic Alzheimer's disease Aβ oligomers. *J Mol Biol* **2012**, *421* (4-5), 517-524.
(19) Lange, A.; Luca, S.; Baldus, M. Structural Constraints from Proton-Mediated Rare-Spin Correlation Spectroscopy in Rotating Solids. *Journal of the American Chemical Society* **2002**, *124* (33), 9704-9705.
(20) Stevens, T. J.; Fogh, R. H.; Boucher, W.; Higman, V. A.; Eisenmenger, F.; Bardiaux, B.; van Rossum, B. J.; Oschkinat, H.; Laue, E. D. A software framework for analysing solid-state MAS NMR data. *J Biomol NMR* **2011**, *51* (4), 437-447.
